# Intermediate Monocytes with PD-L1 and CD62L Expression as a Possible Player in Active SARS-CoV-2 Infection

**DOI:** 10.3390/v14040819

**Published:** 2022-04-15

**Authors:** Elżbieta Rutkowska, Iwona Kwiecień, Krzysztof Kłos, Piotr Rzepecki, Andrzej Chciałowski

**Affiliations:** 1 Laboratory of Flow Cytometry, Department of Internal Medicine and Hematology, Military Institute of Medicine, 04-141 Warsaw, Poland; ikwiecien@wim.mil.pl; 2 Department of Infectious Diseases and Allergology, Military Institute of Medicine, 04-141 Warsaw, Poland; kklos@wim.mil.pl (K.K.); achcialowski@wim.mil.pl (A.C.); 3 Department of Internal Medicine and Hematology, Military Institute of Medicine, 04-141 Warsaw, Poland; przepecki@wim.mil.pl

**Keywords:** monocyte, intermediate monocyte, non-classical monocyte, COVID-19, PD-L1, CD62, TIM-3, convalescent

## Abstract

Monocytes play a role in viral biology, but little is known about the monocyte subpopulation in the course of COVID-19 disease. The aim of the study was the analysis of classical, intermediate and non-classical monocytes with expression of PD-L1 and CD62L, TIM-3 and CD86 molecules in peripheral blood (PB) to distinguish patients with SARS-CoV-2 infection from convalescent patients. The study group consisted of 55 patients with SARS-CoV-2 infection and 51 convalescent patients. The cells were analyzed by flow cytometry. The number and proportion of monocytes were lower in patients with COVID-19 than convalescent patients. We observed a lower proportion of non-classical monocytes in COVID-19 patients than convalescent ones. There was a higher proportion of PDL-1-positive intermediate monocytes in COVID-19 patients than convalescent ones. We noticed a higher geometric mean fluorescence intensity (GeoMean) of PD-L1 on intermediate monocytes in COVID-19 patients than convalescent patients, and a higher proportion of CD62L-positive monocytes in COVID-19 patients in comparison with convalescent ones. We found a higher GeoMean of CD62L on monocytes in COVID-19 patients than convalescent ones. Assessment of PD-L1- and CD62L-positive monocyte subsets may identify patients with a possible predisposition for rapid recovery. The monitoring of monocyte subsets in PB might be a useful test in COVID-19 patients.

## 1. Introduction

Coronavirus disease (COVID-19) is a heterogeneous disease caused by the SARS-CoV-2 virus, usually associated with mild to moderate symptoms such as low fever, dry cough and fatigue [1]. In severe cases, it can lead to acute interstitial pneumonia as well as acute respiratory distress syndrome (ARDS), multiple organ failure or even death [2]. Age, various comorbidities, including diabetes, obesity, lung and cardiovascular diseases, and genetic polymorphisms correlate with a higher risk of respiratory failure [3,4]. Some patients experience a sudden deterioration and the reason for this phenomenon is the so-called cytokine storm caused by an abnormal over-response of the immune system [5]. Defense against SARS-CoV-2 requires an innate immune system with monocytes, granulocytes, dendritic cells (DC) and natural killer (NK) cells, and an adaptive immune system with T and B lymphocytes [6]. 

An increase in inflammatory factors, changes in morphological parameters, the role of lymphocytes in the course of COVID-19 infection, the phenomenon of lymphopenia and the reduction in the number of eosinophils or dendritic cells are well confirmed [7,8,9].

In acute COVID-19 states, a significant decrease in T lymphocytes of both CD4+ and CD8+ cells is observed, with their simultaneous activation [10]. Antibody-secreting plasmablasts dominate among B lymphocytes [11]. There is visible neutrophilia and an increase in neutrophil activation parameters or high neutrophil/lymphocyte ratio (NLR) [12,13]. Cumulative assessment of the above parameters, cytokine profile, leukocyte subpopulation and computed tomography (CT) lung inflammation can help to characterize and differentiate a patient with advanced COVID-19 rather than a single-parameter study [14].

The role of monocytes in COVID-19 disease is not fully understood and not very widely researched. It is known that, along with dendritic cells, monocytes, in addition to being professional antigen-presenting cells (APCs), detect and phagocytose pathogens, mediate leukocyte recruitment, initiate immune responses and regulate inflammation [15,16].

Immunological studies have shown that monocytes are a heterogeneous population and can be divided into three subsets based on the presence and strength of expression of specific surface markers. On the basis of the differences in expression of CD14 and CD16 antigens, the following have been highlighted: classical (CD14++ CD16−), which account for 80–90% of peripheral blood monocytes; intermediate (CD14+ CD16+); and non-classical (CD14−/+ CD16++) monocyte populations [17,18]. The percentages of monocyte subsets may vary with the presence of disease or inflammation [19,20]. The expression pattern of these surface markers is indicative of the functions performed by these populations. CD14 acts as a co-receptor for the Toll-like receptor 4 (TLR4) and participates in lipopolysaccharide signaling (LPS), while the CD16 antigen is identified as a receptor for FcγRIIIa immunoglobulins and participates in innate immunity [21].

Moreover, monocytes express other antigens that influence their function. CD62L, also known as L-selectin, is a cell adhesion molecule playing a role in regulating the recruitment of monocytes to tissue from the blood during inflammation [22]. The induction of critical costimulatory molecules such as CD86 and CD80 on the surfaces of cells such as monocytes combines the innate and acquired immune responses through high antigen presentation capacity and stimulation of CD4+ T cell proliferation [23]. T cell immunoglobulin and mucin-domain containing-3 (Tim-3) is a type I trans-membrane protein acting as a co-inhibitory receptor expressed on IFN-γ-producing T cells, T regulatory cells (Tregs) and innate immune cells, as DCs and macrophages, suppressing their responses upon interaction with their ligand [24]. Tim-3 expression was also confirmed on the surfaces of monocytes and could be used as a potential indicator to evaluate disease severity [25,26].

Other works also show the presence of the programmed death ligand-1 (PD-L1) molecule on the surfaces of monocytes, indicating their role in suppressing the immune response and association with a poor prognosis [27]. It is known that the programmed death receptor 1 (PD-1)/PD-L1 ligand signaling pathway, as an immune checkpoint, has proven to be a promising treatment strategy for various diseases. PD-1 is present in T cells, B cells, antigen-presenting cells (APCs) and in a few other non-lymphoid tissues, and an association of ligands with PD-1 molecules on the T cell promotes immune suppression [28].

In our research, the above-mentioned molecules were selected to characterize the immune status of monocyte populations, and their activation, excitation or depletion in COVID-19 and convalescent patients, by flow cytometrical analysis. 

In our study, we determined the dominant monocyte subpopulations and assessed the differences in immunocostimulatory antigen expression—PD-L1, Tim-3, CD62L and CD86—between patients with positive SARS-CoV-2 infection and convalescent patients. 

## 2. Materials and Methods

### 2.1. Patients

The study group consisted of 55 patients with positive SARS-CoV-2 infection, 51 convalescent patients after COVID-19 disease and 20 healthy controls (HC).

Patients with a SARS-CoV-2-positive test were confirmed by real-time reverse transcriptase–polymerase chain reaction (RT-PCR) assay for nasopharyngeal swab specimens according to the WHO guidelines. The 51 patients were considered convalescent after clinical stabilization and negative test for the SARS-CoV-2 virus twice.

Patients with a SARS-CoV-2-positive test were newly admitted (Department of Infectious Diseases and Allergology, Military Institute of Medicine). 

COVID-19 patients’ characteristics, including age, gender, clinical symptoms, diseases comorbidities and information about saturation, chest X-ray changes, oxygen supplementation and invasive ventilation, are presented in Table 1. The baseline clinical condition on admission was classified as symptomatic unstable with SpO2 at 86% to 98%, and symptomatic unstable with SpO2 ≤ 90% or ARDS. A total of 47 patients had imaged interstitial densities in the lungs by radiological images. Moreover, 51 patients required oxygen supplementation and 3 required invasive ventilation. 

The decision about the treatment regimen was made by the attending physician, taking into account the current knowledge and recommendations of the Polish Association of Epidemiologists and Infectiologists [29]. Throughout the analyzed period, low-molecular-weight heparin at prophylactic or therapeutic doses, dexamethasone in patients receiving remdesivir and oxygen therapy or lopinavir/ritonavir applied in the first period of the disease, antibiotic therapy in case of secondary bacterial infection, oral or intravenous hydration and symptomatic treatment were recommended in patients with respiratory failure, in accordance with national guidelines. From the analyzed patients, five patients were treated in the intensive care unit (ICU). There was no co-infection in the analyzed group of patients. The mean time of hospitalization was 15.8 ± 10 days.

### 2.2. Materials

Peripheral blood (PB) samples were collected in EDTA-K3 tubes (Beckton Dickinson, Franklin Lakes, NJ, USA), from all patients. All evaluated elements were measured on PB samples collected and processed within 2 h of the sample collection by flow cytometry methods using FACS Canto II BD flow cytometry system (Becton Dickinson, Franklin Lakes, NJ, USA).

Samples were collected from 10 May 2021 to 1 December 2021 at the Military Institute of Medicine (Department of Internal Medicine and Hematology, Laboratory of Hematology and Flow Cytometry and the Department of Infectious Diseases and Allergology).

The PB samples used in the study were taken during routine diagnostics and were approved by the Ethics Committee of the Military Institute of Medicine, and all patients gave informed consent (Military Institute of Medicine Ethics Committee number: 47/WIM/2020. Military Institute of Medicine grant number 585. Decision of 4 March 2021, number: 26/W/2021).

### 2.3. Flow Cytometry Analysis

For flow cytometric analysis, 100 µL of PB and 4 µL of specific monoclonal antibodies were added to each cytometric tube for surface marker detection. Cells were stained with fluorescently labeled antibodies for 20 min at room temperature. Erythrocytes were lysed with Pharm Lyse Lysing Buffer (BD Biosciences, Franklin Lakes, NJ, USA) for 10 min. After washing, cells were analyzed within 2 h. For each sample, a minimum of 100,000 events were collected using the FACS Canto II BD flow cytometry apparatus (BD Biosciences). The data were analyzed with DIVA Analysis software v. 8.0.1 (BD Biosciences) and Infinicyt v. 1.8 Flow Cytometry (Cytognos, Salamanca, Spain).

To evaluate the main leukocyte subsets and monocyte subpopulations, we used the following antibodies: 

CD45-V500-C (catalog number 655873, clone number: 2D1, BD Biosciences), CD3-PerCP-Cy5.5 (catalog number: 332771, clone number: SK7, BD Biosciences), CD4-FITC (catalog number: 345768, clone number: SK3, BD Biosciences), CD8-V450 (catalog number: 560347, clone number: RPA-T8, BD Biosciences), CD19-PE-Cy7 (catalog number: 341113, clone number: SJ25C1, BD Biosciences), CD16-APC-H7 (catalog number: 560195, clone number: 3G8, BD Biosciences), HLA-DR-V450 (catalog number: 655874, clone number: L243, BD Biosciences), CD14-APC (catalog number: 345787, clone number: MȹP9, BD Biosciences). 

Using the appropriate combination of the above antibodies, we distinguished the following.

Main leukocyte subsets:

- lymphocytes: CD45+ bright SSClow;
- lymphocytes T: CD45+ bright SSClow CD3+;
- lymphocytes B: CD45+ bright SSClow CD19+;
- NK cells: CD45+ bright SSC low CD3− CD16+;
- neutrophils: CD45+ SSCbright CD16+;
- eosinophils: CD45+ bright SSCbright;
- basophils: CD45+ dim SSClow;
- monocytes: CD45+ bright SSC+ HLA-DR+.

**Figure A1:**
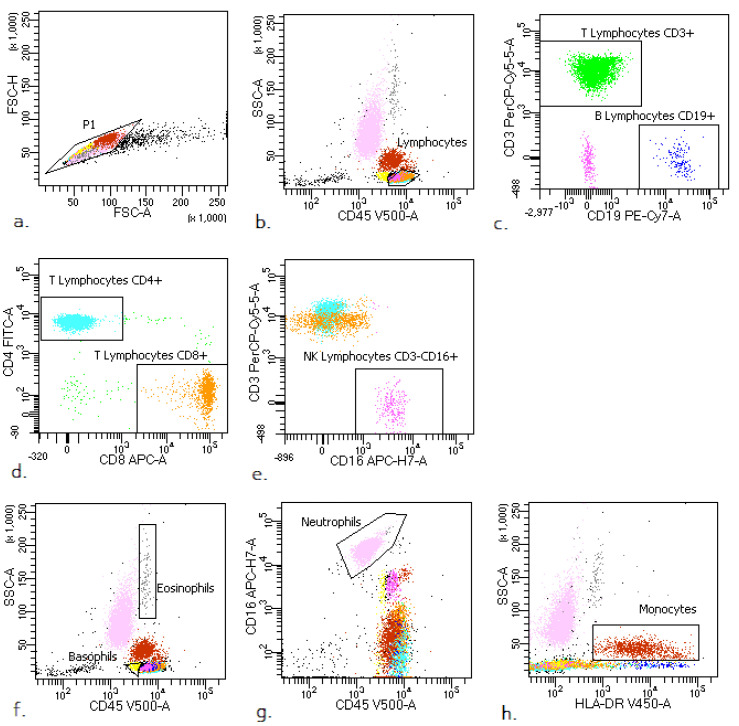
Representative leukocyte subsets’ gating strategy in peripheral blood (PB) of COVID-19 patients. (**a**) FSC-A vs. FSC-H plot: gating the cells that have an equal area and height (P1), thus removing clumps (greater FSC-A relative to FSC-H) and debris (very low FSC). (**b**) SSC-A vs. CD45 plot: selection of lymphocytes based on their SSClow/CD45+ properties. (**c**) CD3 vs. CD19 plot: selection of T lymphocytes (green) and B lymphocytes (blue) based on their CD3+ or CD19+ properties. (**d**) CD4 vs. CD8 plot: selection of CD4 (turquoise) and CD8+ cells (orange) based on their CD4+ or CD8+ properties. (**e**) CD3 vs. CD16 plot: selection of NK cells (pink) based on their CD16+ and CD3− properties. (**f**) SSC-A vs. CD45 plot: selection of eosinophils (grey) based on their SSC-A+bright CD45+ properties. (**g**) CD16 vs. CD45 plot: selection of neutrophils (light pink) based on their CD45+dim CD16+bright properties. (**h**) SSC-A vs. HLA-DR plot: selection of monocytes (red) based on their SSC-A+dim HLA-DR+ properties (the exact antigenic characterization can be found in the text in Section 2, Flow cytometry analysis).

The representative leukocyte subsets’ gating strategy in PB of COVID-19 patients is presented in Figure A1 (Appendix A).

Monocyte subsets:

- Classical monocytes: CD14++ CD16−;
- Intermediate monocytes: CD14+ CD16+;
- Non classical monocytes: CD14−/+ CD16++.

**Figure 1:**
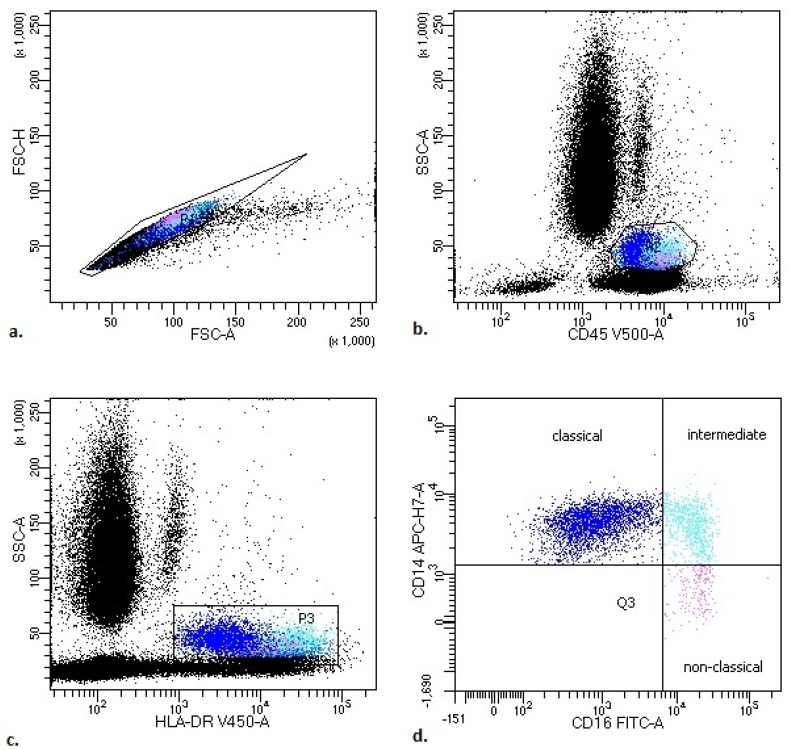
Representative monocyte subsets’ gating strategy in peripheral blood (PB) of COVID-19 patients. (**a**) FSC-A vs. FSC-H plot: gating the cells that have an equal area and height, thus removing clumps (greater FSC-A relative to FSC-H) and debris (very low FSC). (**b**) SSC-A vs. CD45 plot: selection of monocytes based on their SSC+/CD45+bright properties. (**c**) SSC-A vs. HLA-DR plot: selection of monocytes based on their SSC+/HLA-DR+bright properties. (**d**) CD14 vs. CD16 plot: to gate the monocyte subsets—classical (blue), intermediate (turquoise) and non-classical (pink) (the exact antigenic characterization can be found in the text in Section 2, Flow cytometry analysis).

The representative monocyte subsets’ gating strategy in PB of COVID-19 patients is presented in Figure 1. 

We analyzed the expression of PD-L1-PE (catalog number 557524, clone number: NIH1, BD Biosciences) on monocyte subsets and we also assessed the expression of TIM-3-BB515 (catalog number 565568, clone number: 7D3, BD Biosciences), CD62L-PE (catalog number 555544, clone number: -, BD Biosciences) and CD86-APC (catalog number 555660, clone number: -, BD Biosciences) on all monocytes. 

**Figure A2:**
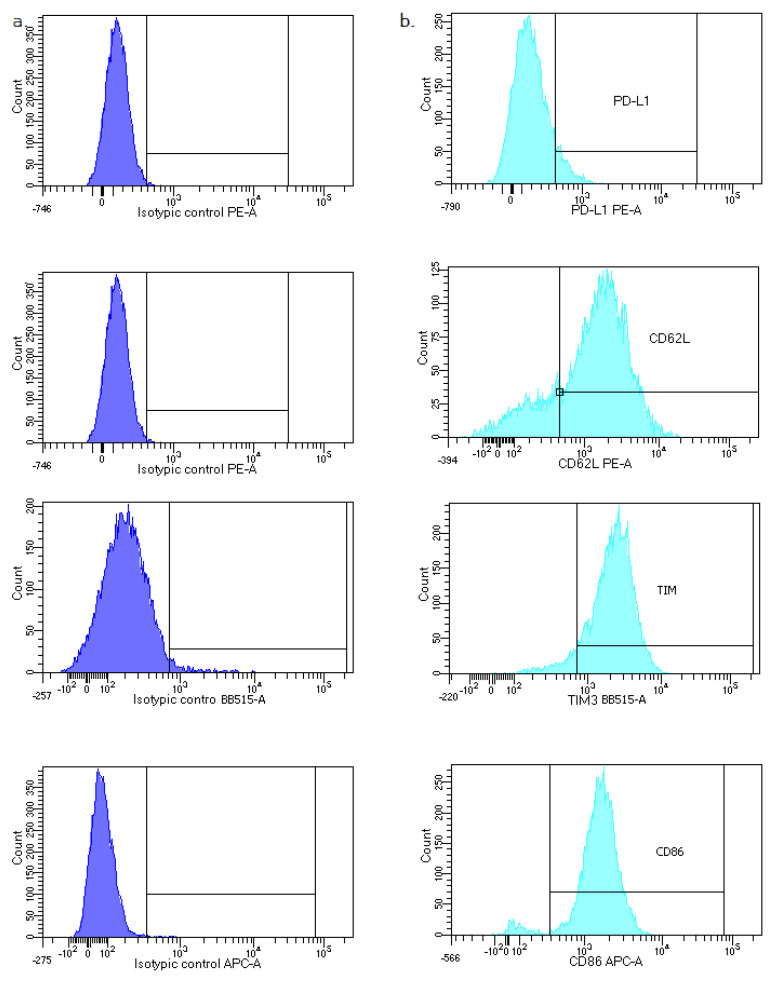
FACS analysis of isotype controls for PD-L1- PE, CD62L-PE, TIM-3-BB515 and CD86-APC. (**a**) Isotype control. (**b**) Representative histograms with PD-L1, CD62L, TIM-3 and CD86 expression in COVID-19 patients.

Isotype control for PD-L1-PE, TIM-3-BB515, CD62L-PE and CD86-APC (PE Mouse IgG1, κ Isotype Control catalog number: 555749, clone: MOPC-21, BD Horizon™ BB515 Mouse IgG1, κ Isotype Control catalog number: 564416, clone: x40, APC Mouse IgG2a, κ Isotype Control catalog number: 555576, clone: G155–178) was applied (Figure A2).

### 2.4. Statistical Analysis

All statistical analyses were performed using the Statistica v. 13.0 software (TIBCO Software, Palo Alto, CA, USA). The results are expressed as means and SDs, and medians with interquartile range (Q1–Q3). For group comparison, the Mann–Whitney U, the Kruskal–Wallis ANOVA test and post-hoc analysis tests were used. For graphic processing, we used Prism GraphPad (Version 7, GraphPad Software, La Jolla, CA, USA). Statistical significance was determined as *p* < 0.05.

## 3. Results

### 3.1. Patients’ Characteristics, White Blood Cell (WBC) Count, Leukocytes and Main Lymphocyte Subpopulation Counts in Study Groups 

The characteristics of the COVID-19-positive investigated group are summarized in Table 1. 

The study groups were compared using the assessment of leukocyte subpopulations by flow cytometry (Table 2). We observed a lower median of absolute number: lymphocytes, including T lymphocytes (both CD4, and CD8), B lymphocytes, eosinophils and basophils, in active COVID-19 patients compared to the convalescent group. 

The median absolute number and median proportion of monocytes were lower in patients with active COVID-19 disease than convalescent patients. The results of all main studied leukocyte subpopulations in active COVID-19 patients and convalescent ones are presented in Table 2. 

### 3.2. Differences in Monocyte Subsets: Classical/Intermediate/Non-Classical and Monocyte Subpopulations with PD-L1 Expression

There are three types of monocytes in PB: the classical monocytes with high expression of the CD14 cell surface receptor and no CD16 expression (CD14++CD16−), the non-classical monocytes with a low/negative level of CD14 expression and co-expression of the CD16 receptor (CD14−/+CD16++) and the intermediate monocytes with expression of CD14 and expression of CD16 (CD14+CD16+). The main monocyte subset in both COVID-19 patients and convalescent patients was classical monocytes. We noticed a lower proportion of intermediate monocytes and the lowest proportion of non-classical monocytes in both groups. 

**Figure 2:**
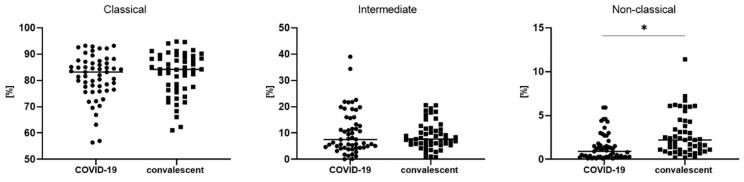
The differences in three types of monocytes—classical, intermediate and non-classical monocytes—between COVID-19 and convalescent patients. Graphs show the median values (−), * *p <* 0.05. Graphs show the median values (Min–Max). Significant differences in the cell proportion between COVID-19 and convalescent patients in the Mann–Whitney U test presented as * (*p* < 0.05).

A significantly lower median proportion of non-classical monocytes in COVID-19 patients compared to convalescent patients (0.9 vs. 2.2%, *p* < 0.05) was observed (Table 3, Figure 2).

In Appendix A, Table A1 shows the differences in monocyte subpopulations relative to HC. We noticed a statistically significant increase in the total monocyte count of convalescent compared to COVID-19 patients and HC. In addition, we noticed a statistically significant increase in classical monocytes and a decrease in non-classical monocytes in COVID-19 patients (active and convalescent) compared to HC. 

**Figure 3:**
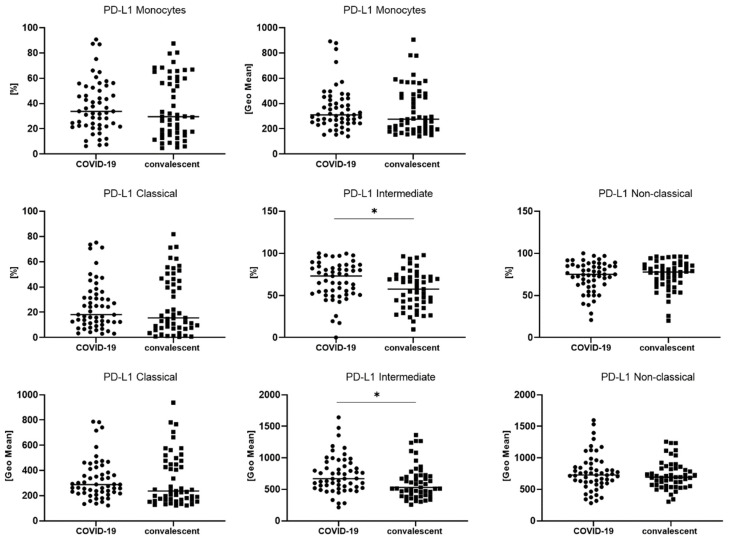
The differences between COVID-19 and convalescent patients for geometric mean expression of PD-L1 on classical, intermediate and non-classical monocytes. Graphs show the median values (−). Significant differences in the cell proportion between COVID-19 and convalescent patients in the Mann–Whitney U test presented as * (*p* < 0.05).

**Figure 4:**
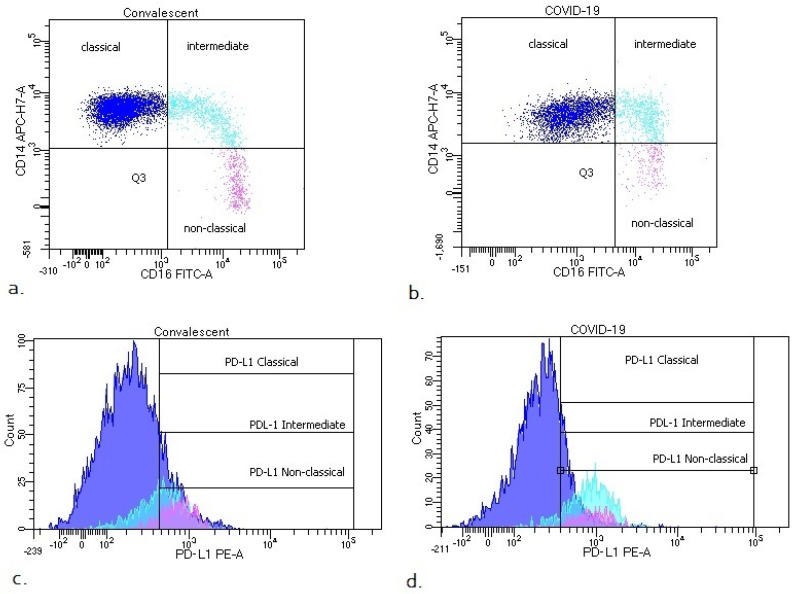
Representative dot plots with monocyte subsets classical, intermediate and non-classical (**a**,**b**) and histograms with monocyte subsets with PD-L1 expression (**c**,**d**) in COVID-19 and convalescent patients.

We also analyzed the median proportion of monocyte subsets with expression of PD-L1 and GeoMean intensity of PD-L1 between COVID-19 patients and convalescent patients (Table 3, Figure 3). We observed a significantly higher median proportion of intermediate monocytes with PD-L1 expression in COVID-19 patients than convalescent patients (73.1 vs. 57.6%, *p* < 0.05). In Figure 3, it is noted that PD-L1-positive monocytes and classic PD-L1-positive monocytes could be divided into two populations ranging from 40 to 80% and 0 to 20% in the convalescent group, but compared to COVID-19, the patients showed no differences. We found a higher GeoMean of PD-L1 on intermediate monocytes in COVID-19 patients than convalescent patients (669 vs. 530, *p* < 0.05). The representative dot plots with monocyte subsets classical, intermediate and non-classical, and histograms with monocyte subsets with PD-L1 expression in COVID-19 and convalescent patients, are presented in Figure 4. 

### 3.3. The Difference in the Number of TIM-3, CD62L and CD86 Postive Monocytes between COVID-19 and Convalescent Patients

**Figure 5:**
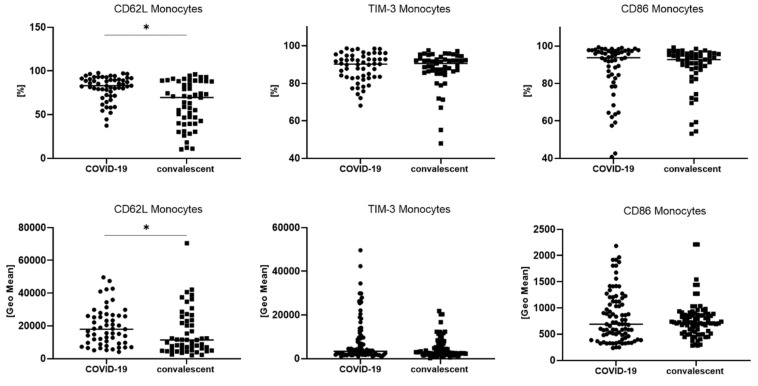
The differences between COVID-19 and convalescent patients for geometric mean expression of CD62L, TIM-3 and CD86 on monocytes. Graphs show the median values (Min–Max). Significant differences in the cell proportion between COVID-19 and convalescent patients in the Mann–Whitney U test presented as * (*p* < 0.05).

We analyzed the median proportion of monocytes with TIM-3, CD62L or CD86 expression and the GeoMean intensity of these markers on monocytes. A significantly higher median proportion of monocytes with CD62L expression (83.0 vs. 69.6% *p <* 0.05) was found. We also noticed a higher GeoMean of CD62L on monocytes in COVID-19 patients than convalescent patients (17,970 vs. 11,362, *p <* 0.05) (Table 4, Figure 5).

## 4. Discussion

Literature data show that monocytes play key roles in severe infections and constitute a first-line cellular response that initiates and promotes a targeted, adaptive immune response. In this study, we analyzed the activation status of monocytes in patients with COVID-19 and convalescents by assessing their subpopulations and specific antigenic pattern using the flow cytometry method.

Most of our patients exhibited typical clinical manifestations for COVID-19 infection, such as fever, cough, dyspnea and fatigue. Acute respiratory failure requiring mechanical ventilation was reported in three patients, representing 5.5% among all COVID-19 patients (Table 1). The group of patients was classified as moderate due to the lack of a severe course of infection. In our study, we found changes between the study groups in the number and percentage of individual leukocyte populations. In the convalescent group, we found higher levels of CD4+ and CD8+ T cells, B lymphocytes and eosinophils and basophils than in patients with active COVID-19 disease, which is in line with the literature data [30,31] and our previous study [12]. 

We observed that monocytes behaved similarly, returning to higher levels in peripheral blood in patients after recovery from COVID-19. The median absolute number and median proportion of monocytes were lower in patients with active COVID-19 infection than convalescent patients. Considering the absolute numbers of monocytes, a statistically significant increase in monocyte number could be seen in convalescent patients compared to active COVID-19 and HC patients. Contrary to our study, an increased number of monocytes was noted in other studies investigating the role of monocytes in COVID-19 infection. Schulte-Schrepping J. et al. found that HLA-DR+high CD11c+high inflammatory monocytes with an interferon-stimulated gene signal were elevated in patients with mild COVID-19 [32]. Other researchers found that proinflammatory monocyte-derived macrophages were abundant in the bronchoalveolar lavage fluid of patients with severe COVID-19 [33]. Otherwise, Qin, S. et al. observed that, in critical patients with COVID-19, the absolute number of total monocytes and CD16− monocytes was significantly decreased but CD16+ pro-inflammatory monocytes were increased compared to healthy controls [34]. According to the authors, during recovery from COVID-19 disease, the count and immune status of monocyte subsets were restored by degrees. Simultaneously, others have shown that a course of severe COVID-19 is associated with reduced expression of the human leukocyte antigen DR isotype (HLA-DR) on CD14+ circulating monocytes, and this was not observed in hospitalized COVID-19 patients without critical illness [35]. The authors also examined the expression of CD38 on monocytes to understand their activation status, and found that the expression of CD38 in the critical group relatively increased as compared with the healthy group.

Therefore, we propose that the standardized flow cytometry assessment of individual monocyte subpopulations and the surface expression of selected antigens can serve as a marker of monocyte immune function in COVID-19 disease and in the course of these infections. 

Flow cytometry analysis indicated a decreased median proportion and count of total monocytes in PB in the COVID-19 group relative to convalescent patients. Some research has suggested that the decreased number of mononuclear cells in the blood of COVID-19 patients may be due to their migration directly to the infected lungs [36].

While the monocytes were divided into subpopulations, the main monocyte subset in both COVID-19 patients and convalescents was classical monocytes. We noticed a lower proportion of intermediate monocytes and the lowest proportion of non-classical monocytes in both groups. It is known that classical monocytes are critical for the initial inflammatory response, which can differentiate into macrophages in tissue, while non-classical monocytes have been widely viewed as anti-inflammatory cells and they are a first line of defense in the recognition and clearance of pathogens [37]. 

However, it turned out that the studied groups differed only in the proportion of non-classical monocytes CD14−/+ CD16++. Compared to HC, we noticed a statistically significant increase in the total monocyte count of convalescent compared to COVID-19 patients and HC. In addition, we noticed a statistically significant increase in classical monocytes and a decrease in non-classical monocytes in COVID-19 patients (active and convalescent) compared to HC. 

In the next step, we examined the expression level of the CD62L antigen on monocytes. Isotype controls were used for markers tested on monocytes to ensure that the isotypes did not cause any background staining in the channels and to monitor compensation/autofluorescence overlap. We observed a higher percentage of CD62L-positive monocytes, as well as a higher density of this antigen on the monocytes’ surfaces (GeoMean value), in COVID-19 patients than in convalescents. Adhesion molecules CD62L are important in monocyte trafficking, enabling their adhesion to the endothelium and transmigration into tissue. Thus, our observation may support the hypothesis that inflammatory cells (including monocytes) migrate to the site of SARS-CoV-2 infection and their numbers are reduced in PB. In addition, our research has shown that the number of non-classical monocytes is significantly lower in COVID-19 patients compared to convalescents, suggesting an important role and high inflammatory potential of these cells. Our results are consistent with reports showing a reduction in the non-classical monocyte subset in viral infections, and in inflammatory or auto-immune diseases, where the decrease in circulation was mainly attributed to tissue migration [38,39,40]. Additionally, the above results also confirm the special role of these cells in responsivity to virus-associated signals [41].

We pointed out the important role of non-classical monocytes in the inflammatory response in COVID-19 disease. We also noticed that the intermediate monocyte subpopulation may inhibit non-classical monocytes at the same time. We examined the expression of the PD-L1 antigen in monocytes and subpopulations of monocytes and found a significant increase in this molecule on intermediate monocytes in COVID-19 patients compared to the convalescent group. The percentage of PD-L1-positive intermediate monocytes was higher, as was the density of this antigen (GeoMean value). An increase in intermediate CD14+ CD16+ monocytes in patients with different clinical severity of COVID-19 in comparison with healthy individuals has been observed before [42,43], but the exact mechanism of this phenomenon has not been elucidated yet. In our study, we showed for the first time PD-L1 expression on intermediate monocytes in COVID-19 patients and convalescent. Other researchers determined PD-L1 expression on monocytes defined as CD14+ (phenotypically similar to the classical monocyte subpopulation). PD-L1 expression on CD14+ monocytes in HC was slightly lower than in our study (10% [44] and 12.7% [45], respectively). Immune checkpoint molecules such as PD-1 and its ligand PD-L1 play an important role in regulating the immune response, and several studies underline the role of PD-1 modulation in infection [46]. There are few and inconclusive data about the significance of PD-L1 dysregulation during SARS-CoV-2 infection. Sabbatino F. et al. demonstrate that serum levels of PD-L1 have a prognostic role in COVID-19 patients and that PD-L1 dysregulation is associated with COVID-19 pathogenesis [47]. Others show that the expression of inhibitory immune checkpoints including PD-1 and PD-L1 on the T cells’ surfaces is enhanced [48]. There are reports showing that SARS-CoV-2 infection impairs the function of mature monocytes by increasing the level of PD-L1 on monocytes [49]. Christensen E. et al. observed that PD-L1 on monocytes increased with COVID-19 severity and in deteriorating patients during the first week of follow-up, whereas in recovered patients, there was a decrease in the expression of PD-L1 [50]. In conclusion, we can indicate that an increase in PD-L1 on intermediate monocytes may contribute to T cell suppression through the PD-1/PD-L1 signaling axis; however, the exact role of the PD-1/PD-L1 pathway in COVID-19 pathology should be investigated in future studies.

When examining the remaining CD86 and TIM-3 markers related to the activation or depletion of monocytes, we did not find significant differences between the groups. El Sehmawy et al. [51] show that healthy people have lower percentages of monocytes expressing CD86 compared to the COVID-19 patients in our study.

We have not found studies in the literature that assess Tim-3 expression on monocytes in COVID-19 patients. There are studies assessing Tim-3 expression in COVID-19 patients but on other cell subpopulations. Varchetta S. et al. [52] have shown that NK cells and CD8+ T cells overexpress T cell immunoglobulin and TIM-3. TIM-3 is a negative regulator of immune cell function; indeed, engagement with its ligands induces T and NK cell exhaustion in different viral infections [53].

Considering the expression of CD86 on monocytes, Carter, M.J. et al. have shown reduced CD86 expression together with elevated levels of IL-1β, IL-6, IL-8, IL-10, IL-17 and IFN-γ in children with multisystem inflammatory syndrome (MIS-C) associated with SARS-CoV-2 infection [54]. Arunachalam et al. [55] also demonstrated a reduction in CD86 and HLA-DR on monocytes and DCs of COVID-19 patients, which was most pronounced in subjects with severe COVID-19 infection. In another study, COVID-19 monocytes exhibited an upregulation of PD-L1 and downregulation of HLA-DR and CD86, which were the hallmarks of the infection [49]. 

We acknowledge that this study is not without limitations. Our experiments were performed on peripheral blood cells, yet many of the innate immune processes may be specific to particular organ microenvironments. However, to our knowledge, this is the first study utilizing monocyte subsets with expression of PD-L1 and monocytes with the CD62L marker, which showed differences between active COVID-19 patients and convalescent patients, and, as such, it provides a background for future research.

Our study shows that the assessment of subtypes of monocytes along with the analysis of immunomodulatory molecules can be significant to assess the course of SARS-CoV-2 infection, and thus in evaluating patients’ recovery and distinguishing active patients from convalescents. 

## 5. Conclusions

In conclusion, our findings show that the assessment of monocyte subsets with PD-L1 expression and analysis of CD62L expression on all monocytes may be critical for predicting the COVID-19 course and identifying patients with a possible predisposition for rapid recovery. This study increases the knowledge of the specific myeloid subsets involved in the pathogenesis of COVID-19 disease and could be useful for the design of therapeutic strategies for fighting SARS-CoV-2 infection.

## Figures and Tables

**Table 1:** Demographic and laboratory data of COVID-19 patients.

	COVID-19 Patients *n* = 55
Sex: f/m (*n*)	21/34
Age (mean ± SD years)	58 ± 15
Women (mean ± SD years)	61 ± 14
Men (mean ± SD years)	59 ± 13
Clinical symptoms (%) (no/yes)	
- fever	12.7/87.3
- cough	23.6/76.4
- dyspnea	25.4/74.6
- respiratory failure	78.1/21.9
Diseases comorbidities (%) (no/yes)	
- diabetes	78.1/21.9
- hypertension	50.9/49.1
- obesity	81.8/18.2
- coronary heart disease	81.8/18.2
- neoplastic diseases	90.9/9.1
Saturation (mean ± SD years)	90.0 ± 6.5
Chest X-ray changes (%) (no/yes)	14.5/85.5
Oxygen supplementation (%) (no/yes)	7.2/92.8
Invasive ventilation (%) (no/yes)	94.5/5.5

Abbreviation: f: female, m: male.

**Table 2:** Differences in the median white blood cell (WBC) count and leukocyte and main lymphocyte subpopulation counts between patients with COVID-19 and convalescent COVID-19 patients. Data expressed as median (Q1–Q3). A * marks statistical significance at *p <* 0.05 (Mann–Whitney U test).

WBC and Study Subpopulation [k/µL]	Patients with COVID-19 Median (Q1–Q3)	Convalescent Median (Q1–Q3)	* *p* < 0.05 Mann–Whitney U Test
WBC	7000 (4640–9020)	7930 (6600–10,470)	* 0.024360
Lymphocytes	1025 (710–1570)	1662 (1170–2199)	* 0.000014
T Lymphocytes	680 (422–1103)	1271 (826–1607)	* 0.000005
CD4 cells	461 (271–675)	775 (559–1117)	* 0.000047
CD8 cells	204 (130–403)	439 (238–567)	* 0.000133
Ratio CD4/CD8	2.0 (1.3–2.9)	2.1 (1.2–2.8)	0.825576
B Lymphocytes	120 (64–216)	172 (120–281)	* 0.016123
NK cells	146 (79–253)	178 (74–300)	0.434570
Neutrophils	5143 (3192–7941)	5319 (4135–7581)	0.457164
Eosinophils	8 (0–38)	72 (18–190)	* 0.000006
Basophils	9 (4–21)	24 (8–54)	* 0.005186
Monocytes	377 (260–454)	536 (399–815)	* 0.000020
% of all leukocytes			
Lymphocytes	14,7 (9.1–28.2)	22.4 (12.8–30.5)	* 0.044030
T Lymphocytes	10.5 (5.5–20.7)	16.9 (8.9–22.2)	* 0.013242
CD4 cells	5.6 (3.5–13.3)	10.5 (5.4–14.7)	* 0.016998
CD8 cells	3.5 (1.8–6.1)	4.9 (3.4–7.1)	* 0.046088
B Lymphocytes	1.7 (1.0–2.6)	2.1 (1.4–3.2)	0.225750
NK cells	2.5 (1.1–4.1)	2.0 (1.0–3.9)	0.492249
Neutrophils	79.4 (63.4–86.9)	68.8 (60.1–79.1)	* 0.015562
Eosinophils	0.1 (0.0–0.8)	1.1 (0.2–2.4)	* 0.000093
Basophils	0.1 (0.1–0.3)	0.3 (0.1–0.6)	0.060189
Monocytes	5.5 (3.8–7.6)	7.0 (4.6–9.1)	* 0.037130

Abbreviation: WBC: white blood count.

**Table 3:** Median proportion of three types of monocytes and monocyte subsets with PD-L1 expression between patients with COVID-19 and convalescent COVID-19 patients. Data expressed as median (Q1–Q3). (* *p <* 0.05 Mann–Whitney U test).

	Patients with COVID-19 Median (Q1–Q3)	Convalescent Median (Q1–Q3)	* *p* < 0.05 Mann–Whitney U Test
All monocytes [% of leukocytes]	5.5 (3.8–7.6)	7.0 (4.6–9.1)	* 0.037130
[% of monocytes]			
Classical monocytes CD14++ CD16-	83.2 (76.5–87.1)	84.2 (76.9–88.8)	0.460992
Intermediate monocytes CD14+ CD16+	7.5 (4.4–15.7)	7.7 (5.6–11.7)	0.904777
Non classical monocytes CD14−/+ CD16++	0.9 (0.3–1.8)	2.2 (1.1–4.3)	* 0.000098
PD-L1+ monocytes [%]	33.8 (22.4–52.5)	29.6 (15.7–60.3)	0.659322
PD-L1+ classical monocytes [%]	18.1 (11.4–34.3)	15.5 (7.5–46.3)	0.659322
PD-L1+ intermediate monocytes [%]	73.1 (52.1–84.4)	57.6 (38.4–71.9)	* 0.007261
PD-L1+ non-classical monocytes [%]	75.0 (62.5–85.7)	77.8 (64.8–86.2)	0.673065
PD-L1+ monocytes [GeoMean]	310 (247–435)	276 (194–476)	0.391353
PD-L1+ classical monocytes [GeoMean]	288 (222–412)	237 (166–477)	0.228186
PD-L1+ intermediate monocytes [GeoMean]	669 (523–919)	530 (407–723)	* 0.015287
PD-L1+ non-classical monocytes [GeoMean]	728 (600–909)	692 (558–832)	0.427183

Abbreviation: GeoMean: geometric mean fluorescence intensity.

**Table 4:** Median proportion and median geometric mean expression of TIM-3, CD62L and CD86 markers on monocytes between patients with COVID-19 and convalescent patients with COVID-19. Data expressed as median (Q1–Q3). (* *p <* 0.05 Mann–Whitney U test).

	Patients with COVID-19 Median (Q1–Q3)	Convalescent Median (Q1–Q3)	* *p* < 0.05 Mann–Whitney U Test
CD62L+ monocytes [%]	83.0 (72.4–91.3)	69.6 (40.1–88.2)	* 0.000107
TIM-3+ monocytes [%]	90.2 (83.3–94.8)	90.6 (86.3–93.2)	0.855115
CD86+ monocytes [%]	93.7 (82.7–97.3)	92.7 (87.3–95.7)	0.387873
CD62L+ monocytes [GeoMean]	17,970 (9645–26,204)	11,362 (5154–23,498)	* 0.020923
TIM-3+ monocytes [GeoMean]	2333 (1715–3197)	2141 (1706–2638)	0.172704
CD86+ monocytes [GeoMean]	724 (483–1188)	725 (539–886)	0.618789

Abbreviation: GeoMean: geometric mean fluorescence intensity.

## Data Availability

Not applicable.

## Appendix A

**Table A1:** Median proportion of three types of monocytes in patients with COVID-19, convalescent patients with COVID-19 and healthy controls (HC). Data expressed as median (Q1–Q3). (* *p <* 0.05 in Kruskal–Wallis ANOVA test and post-hoc analysis).

	Patients with COVID-19 a Median (Q1–Q3)	Convalescent b Median (Q1–Q3)	Control Group c Median (Q1–Q3)	*p* < 0.05 * Group A-B-C ANOVA, Kruskal–Wallis	*p* < 0.05 * between Groups Post-Hoc
**All monocytes [%]**	5.5 (3.8–7.6)	7.0 (4.6–9.1)	6.4 (4.4–7.5)	*p* = 0.1038	-
**All monocytes [k/ul]**	376 (260–453)	535 (399–815)	356 (287–437)	* *p <* 0.0001	* a–b, b–c
**Classical monocytes** CD14++ CD16−	83.2 (76.5–87.1)	84.2 (76.9–88.8)	67.3 (60.7–70.9)	* *p <* 0.0001	* a–c, b–c
**Intermediate monocytes** CD14+ CD16+	7.5 (4.4–15.7)	7.7 (5.6–11.7)	5.2 (3.9–7.2)	* *p* = 0.0472	-
**Non-classical monocytes** CD14−/+ CD16++	0.9 (0.3–1.8)	2.2 (1.1–4.3)	14.9 (12.5–16.8)	* *p <* 0.0001	* a–b, a–c, b–c
